# Expression of CD31, CD34, and smooth muscle actin (SMA) in endothelial cells of dental pulp vessels

**DOI:** 10.17305/bb.2023.9988

**Published:** 2024-08-01

**Authors:** Ana Tenyi, Aleksandra Milutinović, Lidija Nemeth

**Affiliations:** 1 Department of Dental Diseases and Normal Dental Morphology, Medical Faculty University of Ljubljana, Ljubljana, Slovenia; 2 Institute of Histology and Embryology, Medical Faculty University of Ljubljana, Ljubljana, Slovenia

**Keywords:** Dental pulp, endothelial cells, cluster of differentiation 31 (CD31), smooth muscle actin (SMA), cluster of differentiation 34 (CD34)

## Abstract

The dental pulp is a highly vascularized and innervated loose connective tissue surrounded by hard dental tissues—enamel and dentine. With the primary dentin formation and the closure of the root apex, the conditions in the dental pulp change and pulp tissue compliance are reduced. Endothelial cells of pulpal blood vessels are highly differentiated and are capable of adaptation to changes in the environment. We aimed to evaluate the phenotypic plasticity of endothelial cells of pulpal blood vessels in permanent premolars with open (*N* ═ 6) or closed root apex (*N* ═ 30). The pulp tissue was stained with hematoxylin–eosin (HE) for the histological analysis, and immunohistochemically for a cluster of differentiation 31 (CD31), a cluster of differentiation 34 (CD34), and for smooth muscle actin (SMA) to detect vessels with CD31, CD34, and SMA positive endothelial cells. We used the student’s *t*-test and Pearson correlation test for the statistical analysis. We found a significantly higher percentage of the vessels with CD31 (*P* ═ 0.005) and CD34 (*P* ═ 0.000) positive endothelial cells in the group of teeth with closed root apex compared to the group with open apex. A significant positive correlation between SMA positive and CD31 positive vessels (*P* ═ 0.003) and between CD31 positive and CD34 positive vessels (*P* ═ 0.031) was also found. We conclude that the endothelial cells of dental pulp vessels express a small amount of CD31, but have a pronounced expression of SMA and CD34, which indicates their progenitor potential and contractile ability.

## Introduction

The tooth is composed of a hard outer layer of enamel and dentine, encasing a highly vascularized and innervated dental pulp made of loose connective tissue. Pulp tissue is normoxic if the partial pressure of oxygen is around 23.2 mmHg [1, 2]. A rich network of blood vessels enables sufficient oxygen pressure. The volume of blood in the pulp is similar to tissue in breast tumors, representing about 3% of the wet weight [3, 4].

The wall of the blood vessels in the dental pulp is formed by endothelial cells that lie on the basement membrane, pericytes, and smooth muscle cells [1]. Endothelial cells are highly differentiated and depend on the tissue supplied by the vessels in terms of morphological, functional, and metabolic characteristics [5]. In addition to differences from organ to organ in structure, function, metabolic properties, response to growth factors, and antigenic composition, endothelial cells also differ within different organs or organ systems in vessel size (macro and microvessels) [6]. In normal conditions, endothelial cells maintain their phenotype, but they are also capable of adapting to the environment, which is called phenotypic plasticity [5]. The closure of the root apex causes the reduction of pulp tissue compliance and the environmental conditions in the dental pulp change. The primary dentin is no longer being built, and the demands for collagen production and supply of calcium salts are reduced to the level of basal metabolism, indicating changes in blood circulation [7].

In this study, we aimed to evaluate the variations in endothelial cells of pulpal blood vessels in teeth with open or closed root apex. Therefore, the pulp tissue was stained immunohistochemically with anti-cluster of differentiation 31 (CD31), anti-cluster of differentiation 34 (CD34), and anti-smooth muscle actin (SMA). Cluster of differentiation (CD) refers to proteins found on the surface of cells. Each protein that enables the identification and characterization of cellular phenotypes has its number (e.g., CD31) [8].

Endothelial cells constitutively express platelet endothelial cell adhesion molecule 1 (PECAM-1) or CD31. The immunohistochemical detection of CD31 is often used for the demonstration of endothelial cells in tissue sections [9]. CD31 is a transmembrane protein composed of extracellular, transmembrane, and intracellular portions. The extracellular portion has two domains (IgD1 and IgD2) that mediate homophilic CD31–CD31 interactions between endothelial cells and leukocytes, as well as between endothelial cells. In the process of adhesion at the endothelial cell-to-cell borders, CD31 functions as a regulator of vascular permeability and a main endothelial mechano-sensor [10]. CD31 is known to be involved in the processes of downstream inhibitory signaling to regulate many processes, such as platelet and leukocyte activation and adhesion, endothelial cell-to-cell adhesion, and angiogenesis [11, 12].

CD34 is a transmembrane protein expressed on hemopoietic and endothelial progenitor cells [13]. It serves as a signaling molecule that maintains some cells persisting in a phenotypic plastic state, enabling them to form new blood vessels in adult tissues [13, 14].

SMA, a known marker for vascular smooth muscle cells [15], is also expressed in pericytes [16] and endothelial cells of cardiac microvessels [6].

## Materials and methods

### Patients and extracted teeth

Thirty-six permanent upper and lower premolars of ten patients, aged from 12 to 22 years, were included in the study. The teeth were extracted due to orthodontic indications.

### Tissue samples and staining

Tissue samples of dental pulps were prepared according to the protocol described in detail in our previous article [17].

Immediately after extraction, the apical foramen in each root was measured using an endodontic hand file instrument with a known standardized dimension to assess the diameter [17]. The apical third of the root was then drilled off to allow better penetration of the formalin into the pulp tissue and then fixed for 24 h [18]. The tooth was split longitudinally into two halves with a surgical chisel and re-immersed in the formalin for another 48 h. The pulp was gently removed from the dentinal wall with surgical dressing forceps, dehydrated in alcohol, immersed in xylene, and then embedded in paraffin. The tissue samples of dental pulp were sliced into 4.5-µm thick longitudinal step serial sections. The thickness of the step was 20 µm. Sections were stained with hematoxylin–eosin (HE), and immunohistochemically for the detection of endothelial cells (anti-CD31; 1:15, DACO, Glostrup, Denmark), endothelial cells persisting in a phenotypic plastic state (anti-CD34, 1:20, DACO, Glostrup, Denmark), and smooth muscle cells (SMC; anti-smooth muscle actin; 1:100, Cell Marque) following the manufacturer’s instructions as described previously [19, 20].

### Teeth with open and closed root apices

After the measurement of apical foramens, teeth samples were divided into two groups according to their diameter: the group with open root apex (*N* ═ 6) and the group with closed apex (*N* ═ 30). The apices with less than 0.015 mm diameter were defined as closed, and those with more than 0.04 mm as open [17, 21]. None of the teeth had the diameter of apices between 0.015 and 0.04 mm.

### Image analysis and evaluation of the expression of CD31, CD34, and SMA in endothelial cells

Image analysis was performed under a light microscope (Nikon Eclipse E 400), a camera (Nikon digital sight DS-M5), and NIS elements version 3-D computer program. The analysis was performed on three slices of the dental pulp in sagittal orientation in four regions of interest (ROI – 611 × 460 µm) at the objective magnification of 20×. The two ROIs were captured in the crown portion of the dental pulp and two in the root. In the ROI, the blood vessels with and without the expression of CD31, CD34, and SMA in endothelial cells were counted and expressed in percentages (%). The positive expression of the markers was defined as positive staining of at least half of the luminal surface of the vessel.

### Ethical statement

The study received approval from the Slovenian National Medical Ethics Committee under protocol number 0120-415/2020/6. Before clinical procedures, all invited participants received and signed proper information on the informed consent form.

### Statistical analysis

Analysis was conducted using Microsoft Excel 2010 and Statistical Package for Social Sciences SPSS-20.

The power of the study, set at 0.8, and the significance of the *P* value at < 0.01, showed that the appropriate sample size was 19 dental pulps.

We calculated the average values ± standard deviation (SD) of the percentage of the vessels with CD31, CD34, and SMA positive endothelium for both the open (*N* ═ 6) and closed apex (*N* ═ 30) groups.

The student’s *t*-test (*P* < 0.05) was used to evaluate the statistical significance of the differences between both tested groups. Although the samples were not equal in size, the variances in both groups were equal (Levene’s test).

The relationships between the percentage of vessels with SMA, CD31, and CD34 positive endothelial cells were tested by the Pearson coefficient of correlation (*P* < 0.05).

## Results

### Tissue samples

Histological analysis of dental pulps stained with HE clearly showed the odontoblastic and sub-odontoblastic zones at the periphery followed by a cell-free zone (Weil zone) toward the central part of the pulp, the cell-rich layer (Höhl zone) with loose connective tissue. The odontoblastic layer was often separated or torn from the dentinal wall during the procedure of pulp tissue isolation.

**Figure 1.**
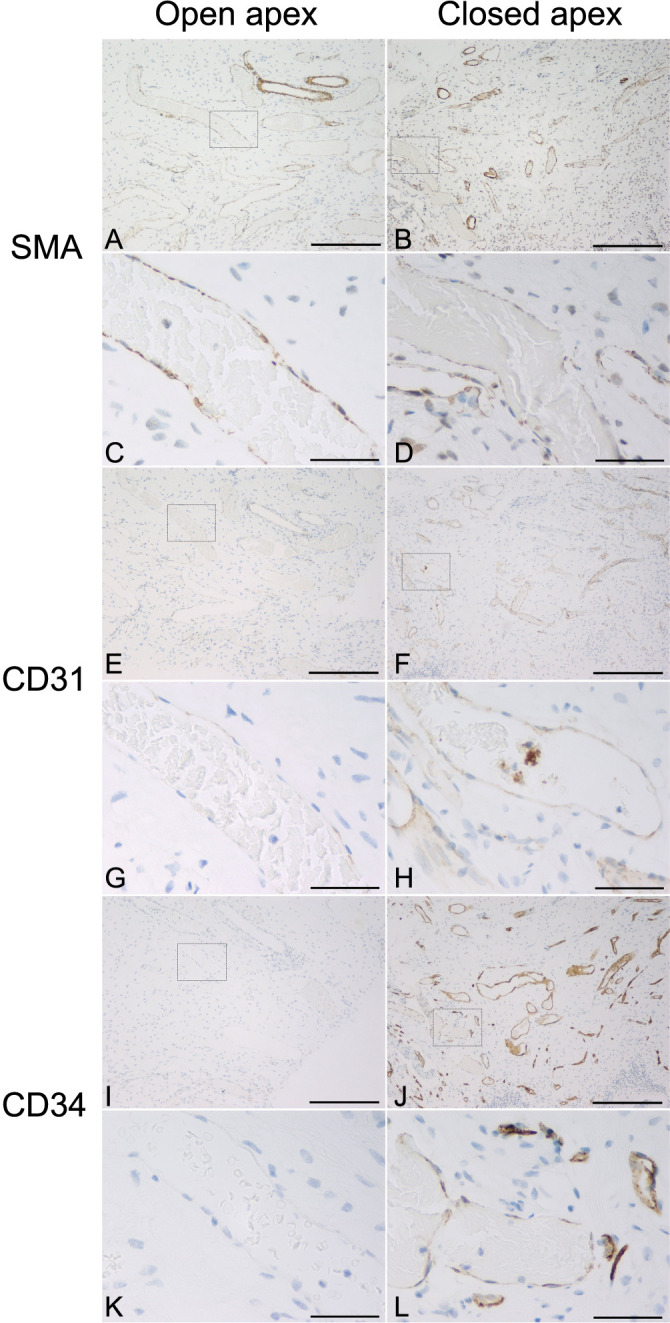
The immunohistochemical staining for (A–D) SMA, (E–H) CD31, and (I–L) CD34 of the dental pulp with open (A, C, E, G, I, K) and closed root apex (B, D, F, H, J, L); Objective magnification ×10, bar ═ 300 µm (A, B, E, F, I, J); Objective magnification ×60, bar ═ 50 µm (C, D, G, H, K, L). SMA: Smooth muscle actin; CD31: Cluster of differentiation 31; CD34: Cluster of differentiation 34.

The immunohistochemical staining for CD31, CD34, and SMA showed that the pulp tissue is well vascularized. Most vessels with positive endothelium had weak CD31 expression, weak to moderate SMA expression, and moderate to strong CD34 expression. Many vessels, especially venules, have fragile walls consisting only of an endothelial layer but have a wide lumen (Figure 1).

The main arterioles and venules in the central part of the dental pulp tissue ran parallel to the long axis of the tooth. Blood vessels branched off many times at right angles. These branches often had a greater caliber than that of the mother vessel and often ran in transversal directions.

In coronary pulp, especially in the upper part, the blood vessels extensively branched off to the dense capillary bed.

### Comparison of CD31, CD34, and SMA expression in endothelial cells between both groups

**Figure 2.**
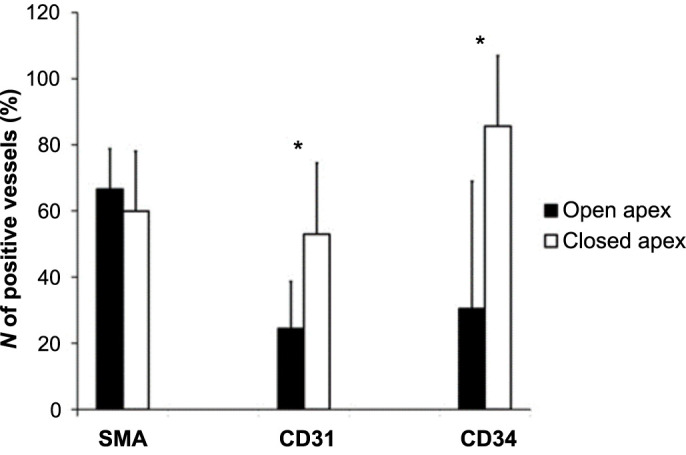
**Percentage of vessels with SMA, CD31, and CD34 positive endothelial cells in the group with open and closed root apex**. Note that there were significant differences in CD31 and CD34 positive endothelial cells between both groups (*significantly different, student’s *t*-test, *P* < 0.05). SMA: Smooth muscle actin; CD31: Cluster of differentiation 31; CD34: Cluster of differentiation 34.

The dental pulp samples were divided into the group of teeth with closed (*N* ═ 30) and with open root apex (*N* ═ 6). We found a significantly higher percentage of the vessels with CD31 and CD34 positive endothelium in the group with closed apex (52.97% ± 21.51 and 85.65% ± 21.27, respectively) compared with the group with open apex (24.41% ± 14.29 and 30.44% ± 38.54, respectively), (*P* ═ 0.005; *P* ═ 0.000). There were no significant differences in the percentage of vessels with SMA positive endothelial cells between both groups (*P* ═ 0.442; closed apex: 59.94% ± 18.13, open apex: 66.62% ± 12.13) (Figure 2).

### Correlation between percentages of vessels with SMA, CD31, and CD34 positive endothelial cells

The average percentage of vessels with SMA, CD31, and CD34 positive endothelial cells in all 36 dental pulps was 61.09% ± 17.82, 48.21% ± 23.00, and 76.45% ± 31.98, respectively.

**Figure 3.**
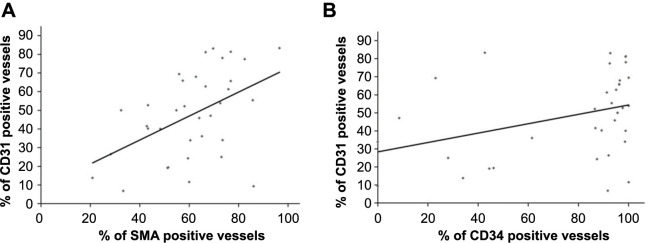
(A) Linear correlation between percentage of vessels with SMA positive endothelial cells (SMA positive vessels) and percentage of vessels with CD31 positive endothelial cells (CD31 positive vessels) (*r* ═ 0.481, *P* ═ 0.003, Pearson correlation); (B) Linear correlation between percentage of vessels with CD34 positive endothelial cells (CD34 positive vessels) and percentage of vessels with CD31 positive vessels (*r* ═ 0.361, *P* ═ 0.031, Pearson correlation). SMA: Smooth muscle actin; CD31: Cluster of differentiation 31; CD34: Cluster of differentiation 34.

We found a significant positive correlation between SMA and CD31 positive vessels (*r* ═ 0.481, *P* ═ 0.003) and between CD31 and CD34 positive vessels (*r* ═ 0.361, *P* ═ 0.031), but not between SMA and CD34 positive vessels (*r* ═ –0.106, *P* ═ 0.546) (Figure 3).

## Discussion

In this study, we evaluated changes in the expression of CD31, CD34, and SMA in the endothelium of blood vessels in dental pulps of teeth with open or closed root apex. We found significantly more vessels with CD31 and CD34 positive endothelial cells in the group with closed apex than in the group with open apex, and no significant differences in SMA expression between the groups. There were only six teeth in the open apex group, which is the main limitation of our study. The teeth of ordinary clinical patients undergoing orthodontic treatment were included in the study, so we were unable to select teeth based on the patient’s age or open/closed apex.

CD31 is a well-known marker for endothelial cells and it is produced at a constant rate in both early and mature endothelial cells [22]. Cytokines do not affect its expression [9]. In most tissues, CD31 is well expressed in endothelial cells [23], but not in the dental pulp. A study performed on 36 upper first premolars showed that CD31 expression was weak in as many as 34 teeth [24]. In our study, CD31 expression was also mild, where 48% of vessels had CD31 positive endothelial cells, 53% of which were CD31 positive vessels in pulps with closed root apex and only 24% with an open apex. CD31 plays a role in many signaling, regulatory, and activation processes, including endothelial cell–cell adhesion and angiogenesis [11, 12]. Our previous study showed that the volume density of vessels in the closed root apex group was non-significantly higher than that in the group with an open apex [17]. Another group of researchers measured blood flow in the dental pulp at different stages of root development. They discovered that in teeth with a previously and recently closed root apex, there was a significantly greater blood flow than with an open root apex [25]. In comparison to other tissues, the pressure in the arterial system of the dental pulp enclosed in hard dental tissues is lower, and the pressure in the veins is higher, since the resistance in the pulp vessels is weak, and the resistance outside the pulp on the arterial and venous side is considerable [7]. Conditions in the pulp are likely to be very constant and might affect the expression of intercellular junctions between endothelial cells and the expression of CD31, which is located in the basal portion of the lateral plasma membrane [26].

A similar pattern of expression in endothelial cells as for CD31 was also discovered for the expression of CD34; notably, 86% of vessels with CD34 positive endothelium in the closed root apex group and 30% with an open apex. We also found a positive correlation between vessels with CD31 and CD34 positive endothelial cells.

A study published in 2003 showed that the vascular endothelium of deciduous and permanent teeth in young people expresses CD34, but with aging, CD34 expression decreases [14]. A pronounced expression of CD34 in 16–18 years old people was also confirmed by a study from 2006 [27]. CD34 is a transmembrane signaling protein expressed on hematopoietic and endothelial progenitor cells, as both types of cells are derived from the same progenitor—the hemangioblast. CD34 is known to maintain endothelial cells in a state of phenotypic plasticity. This process occurs in vasculogenesis and angiogenesis. Angiogenesis is a process where new vessels are formed as buds from already existing vessels, while vasculogenesis is formed directly from angioblasts of mesenchymal origin [14, 27]. Also, in our study, we showed intense expression of CD34 in young adults—we found that 86% of vessels were CD34 positive in the group with closed root apex. However, not all vessels were CD34 positive; as many as 14% were CD34 negative. Unlike our expectations, the open root apex group had only 30% CD34 positive vessels. Only certain vessels may have the potential to sprout new vessels. In the group with an open root apex, these potential CD34 positive vessels have not yet budded nor branched, while in the group with a closed apex, they have already branched and thus are more numerous.

In our study, we observed that some endothelial cells in the dental pulp express SMA. Specifically, 61% of vessels were SMA positive, 60% of which were in the closed apex group, and 67% were in the open apex group. An in vitro study from 1999 showed that porcine cardiac microvascular endothelial cells expressed SMA, whereas coronary artery and endothelial cells of the aorta, which are significantly larger vessels, did not express SMA [6]. In our research, we obtained a similar result: the dental pulp contained only small blood vessels, which were often SMA positive.

Vascular stem cells derived from the human umbilical cord, however, simultaneously express SMA, CD34, and CD31 [28]. Since the endothelial cells in the dental pulp in our study also express all three markers, it indicates their possibility of differentiation into endothelial or smooth muscle vessels and/or expressing the properties of both cell types.

We observed weak expression of CD31 in pulp vessels, expression of SMA, and strong expression of CD34 in endothelial cells. The loss of CD31 and the expression of SMA indicate a process where the endothelial cells lose the properties of the epithelium and gain the properties of the mesenchyme (connective cells), known as an endothelial–mesenchymal transition [5]. However, we observed a positive correlation between vessels with endothelial cells expressing CD31 and those expressing SMA, indicating that the endothelial cells did not lose epithelial properties but acquired contractile properties. Unfortunately, we did not double label so we cannot claim that it is the same endothelial cell containing both CD31 and SMA, but given the localization of both labels, we can claim that we were observing endothelial cells that expressed CD31 as well as SMA. The vessels in the pulp have very thin walls and wide lumen. This phenomenon is possibly due to the fact, that vessels need to possess contractile properties to ensure the blood flow. One possible explanation is also that the dilated lumen is due to the effect of the blood pressure on the thin vascular walls. Arterioles have smooth muscle cells in the media, while venules and capillaries have only pericytes. Pericytes, despite their contractile abilities, may not provide adequate blood flow or sufficient strength to the vessel wall. Endothelial cells can adapt to various demands of the environment, and possibly, when the blood flows slow down, they detect it with mechano-sensor and begin to express SMA, thereby gaining the ability to contract. At the same time, the junctions between endothelial cells could be strengthened, as this phenomenon is reflected in the higher expression of CD31.

## Conclusion

We conclude that endothelial cells in the vessels of the dental pulp express a small amount of CD31, but higher levels of SMA and CD34, which indicates their progenitor potential and contractile ability.

## Data Availability

Authors will send full data on request.
